# Tracing individual experiences to systemic challenges: the (re)production of GBV in migrant women’s experiences in Canada

**DOI:** 10.3389/fsoc.2025.1528525

**Published:** 2025-03-04

**Authors:** Busra Yalcinoz-Ucan, Evangelia Tastsoglou, Myrna Dawson

**Affiliations:** ^1^GBV-MIG Canada Research Program, Saint Mary’s University, Halifax Regional Municipality, NS, Canada; ^2^Department of Sociology, Saint Mary’s University, Halifax Regional Municipality, NS, Canada; ^3^Department of Political Science and Global Development Studies, Saint Mary’s University, Halifax Regional Municipality, NS, Canada; ^4^Department of Sociology and Anthropology, University of Guelph, Guelph, ON, Canada

**Keywords:** gender-based violence (GBV), migration, continuum of violence, migrant and refugee women, intersectionality, Canada, intimate partner violence

## Abstract

This study examines the experiences of migrant women survivors of gender-based violence (GBV) in Canada, focusing on their processes of disclosing violence and seeking help. It explores a range of migration-related factors and circumstances that shape migrant women’s responses to violence while also aiming to reveal how migration contexts determine system-and structural-level responses to GBV, which are then traced back to women’s individual experiences and responses. Based on 17 in-depth interviews with migrant women and using a situated intersectionality perspective, our findings demonstrate first how GBV in migration is uniquely shaped and (re)produced by precarity, rooted in structural, socioeconomic, and legal conditions that translate into heightened vulnerability at the individual level. We showed that migration contexts increased women’s vulnerability to GBV, as perpetrators exploited precarity to manipulate and control women, illustrating the continuum of precarity-GBV. Secondly, this manipulation, controlling behaviors, and abuse of migrant women by perpetrators are enabled by migration policies and practices that give rise to their precarity. Additionally, our participants reported a lack of supportive social networks, which, in combination with the fear of cultural stigmatization, created a double bind hindering their processes of seeking safety. Furthermore, systemic responses to migrant women experiencing GBV were found to be inadequate, with discriminatory and negligent attitudes in healthcare, police, and legal systems. This is the continuum of systemic-individual level violence. Our findings enhance both the theoretical and empirical understanding of the continuum (i) between precarity and GBV and (ii) between systemic and individual forms of GBV in migration contexts, where precarity exacerbates GBV, and vice versa, creating a vicious cycle that deepens individual experiences of vulnerability, while the systemic and structural forms of violence contribute/(re)produce individual experiences of GBV.

## Introduction

Migration is considered one of the critical life experiences that can create unique opportunities for rebuilding better lives, but it can also lead to multiple intersecting risks and challenges for families and individuals (Wachter et al., 2021). Increased vulnerability to gender-based violence (GBV) is highlighted as a significant risk that migrant women may face during the course of their migration journeys (Freedman et al., 2022a; Jayasuriya-Illesinghe, 2018). Studies primarily indicate that GBV can be experienced before migration in countries of origin; however, it can also result from migration itself, as women may face heightened risks of violence both before and after their arrival (Freedman et al., 2022a; Tastsoglou and Nourpanah, 2019). Migratory risks may stem from a variety of factors commonly faced by migrant families and individuals, including significant changes in financial and living conditions, precarious migratory status, loss of social networks and supports, and challenges related to social integration (Alaggia et al., 2009; Aujla, 2021; Hulley et al., 2023; Okeke-Ihejirika et al., 2020; Menjívar and Salcido, 2002). Additionally, numerous barriers such as the challenges of learning new languages, limited knowledge of systems and structures in host countries, or encountering clashing cultural norms and beliefs further hinder migrant women’s ability to disclose violence and access support services (Ahmad et al., 2009; George and Rashidi, 2014; Guruge and Humphreys, 2009; Hyman et al., 2006; Hulley et al., 2023; Wachter et al., 2021).

Migration and GBV scholars address the gendered social and economic inequalities faced by migrant women in host countries, alongside the limited protective mechanisms available to them, contributing to migrant women’s vulnerabilities to GBV (Freedman et al., 2022a,b; Guruge et al., 2012; Jayasuriya-Illesinghe, 2018; Singh, 2010). These societal and structural aspects, however, are less commonly investigated and understood in the existing literature. In both academic and public discourses, GBV against migrant women is often perceived and conceptualized within culture-based frameworks, especially when examining racial and ethnic minority women’s experiences of GBV and their so-called “reluctance” to disclose violence and seek support. Furthermore, while a significant number of studies focus on investigating the reasons for low levels of disclosure and help-seeking among migrant women, the research knowledge regarding what responses and reactions they encounter when they do disclose violence and ask for help remains relatively scarce.

Our study contributes to the existing literature by arguing that GBV in migration cannot be solely viewed in “culturalist” terms, but the systemic and structural factors should be dissected and understood (e.g., Freedman et al., 2022b; Jayasuriya-Illesinghe, 2018; Jiwani, 2005; Reilly et al., 2022; Sokoloff and Dupont, 2005). Based on the experiences of seventeen migrant women in Canada, we primarily investigate how migration-related factors and processes shaped their experiences of GBV, particularly regarding the decisions and actions they took to ensure their safety. Broadly drawing upon the concept of “continuum” by Kelly (1987) within a “situated” intersectionality framework (Yuval-Davis, 2015), we first aim to trace the continuum from individual experiences of violence to systemic and structural challenges faced by migrant women. In doing so, we examine how violence is enacted in a cyclical and interconnected manner, both at the level of women’s interpersonal relationships and at the systemic-structural level. Our second goal is to identify and examine the continuum between precarity and GBV in our participants’ lives. We aim to understand how this continuum shapes migrant women’s responses to GBV and restricts their decision-making power in migration contexts. Precarity refers to multilevel vulnerabilities surrounding migrant women’s lives generated by intersecting socioeconomic, structural, and community-level factors (Tastsoglou, 2022; Reilly et al., 2022). Thus, we specifically aim to investigate how these interconnected factors in migration contexts hinder migrant women’s processes of disclosing violence and asking for help. Furthermore, we examine the range of responses migrant women received from both their communities and formal service structures (e.g., the police or healthcare services) when they disclosed GBV and sought assistance. By doing so, we aim to reveal the continuum between precarity and GBV as experienced by our participants.

Building on these primary goals, our study focuses on migration and GBV in the Canadian context. Canada is well-known for its rapidly growing migrant population. In 2021, more than one-quarter (27%) of the women population were reported as migrants (Canada at a Glance, 2022), with a majority coming from Asia, the Middle East, and Africa. This high proportion of migrant women —considering that most are from racial and ethnic minority communities— reveals the importance of studying the risks and vulnerabilities encountered throughout the migration processes. Canadian scholars highlight the need for further research on the complexities of economic, social, and political determinants of migrant women’s experiences of GBV in Canada, particularly the unique forms of violence and the contextual factors surrounding these experiences (Aujla, 2021; Jayasuriya-Illesinghe, 2018; Tastsoglou et al., 2022). Thus, our study contributes to the existing literature by offering a more comprehensive understanding of the multilevel challenges many migrant women encounter.

In the following sections, we first lay out the theoretical understanding we rely on in our conceptualization of GBV and migration. Then, we briefly discuss the previous literature on the topic. Lastly, based on the relevant studies we present a brief analysis of state and policy responses to GBV and migration in Canada. This section will be followed by the presentation and discussion of our findings.

## An intersectional continuum of violence approach to migration and GBV

The intersectionality approach not only enables an understanding of how power and resources are unevenly distributed within societies, creating hierarchies of positionalities for individuals and groups but also of how these hierarchical positionalities intertwine and cross-sect, multiplying the effects of inequalities and vulnerabilities for those affected by them (Yuval-Davis, 2015; Reilly et al., 2022). More specifically, Yuval-Davis’s situated intersectionality perspective addresses how such simultaneous positionings shape individuals’ experiences of disempowerment and marginalization across structural, cultural, and interpersonal contexts (Cole, 2009; Sokoloff and Dupont, 2005). In our study, we heuristically apply such a “situated” understanding of intersectionality to examine migrant women’s lived experiences of GBV. Specifically, we use the intersectionality framework to analyze the context(s) in which individuals and groups are situated and how these social, political, and material contexts dynamically restrict or facilitate people’s access to power and resources (Freedman et al., 2022a). This analysis enables us to illustrate the inherent role of cultural and institutional responses and practices in shaping the conditions of vulnerability in migrant women’s lives. Therefore, we consider this framework crucial and necessary for understanding the range of the continuum of violence and its impact on migrant women’s experiences (Sisic et al., 2024).

Our point of departure in this study is that migration and GBV can create complex circumstances of vulnerability in women’s lives (Rubini et al., 2024: Tastsoglou, 2023; Tan and Kuschminder, 2022). These circumstances are shaped by how societal structures and systems respond to women’s diverse needs and interests (Freedman et al., 2022b). Thus, we conceptualize vulnerability not as an “inherent” characteristic of individuals or groups but as a constantly shifting experience determined by the complexity of individual-society-system interactions. This aligns with the “precarity” approach defined by Reilly et al. (2022, p. 36), which refers “not to permanent individual or group identities but to precarious situations/states produced by such structures and discursive practices.” In this study, combining intersectionality with Kelly’s continuum of violence approach, we view GBV as perpetuated in a continuum of precarity-GBV in migrant women’s lives —precarity that is shaped by gendered power relations and systemic inequalities. This perspective first allows us to identify unique forms of violence experienced by migrant women, which often remain invisible, underrecognized, or normalized in their everyday lives (Kelly, 2012; Sisic et al., 2024). It also demonstrates how harm can be enacted across systemic, structural, cultural, and interpersonal contexts, as each of these interconnectedly determines women’s access to power and resources (Acquadro-Pacera et al., 2024; Rubini et al., 2024; Tastsoglou, 2022).

Finally, conceptualizing GBV in a “continuum” with precarity enables an understanding of how violence can be experienced at any point in time and space during the course of women’s migration journeys. This perspective reveals how “stages” of violence and “safety” (or non-violence) blend together and cannot be straightforwardly located or categorized (Kelly, 1987; Harris and Vitis, 2020). This is also how we conceptualize the relationship between migration and GBV: we ask what particular migration-related dynamics, situations, and contexts interact with or lead to women’s experiences of GBV. In this context, we argue that migrant women, depending on their social location(s) and particular circumstances, can be vulnerable to GBV on different levels in quite distinct ways. Therefore, our analysis seeks to reveal how GBV in migration contexts emerges from precarity —understood as the structural, legal, and socioeconomic conditions that translate into vulnerability at the individual level.

## Research on migrant women’s experiences of GBV

A significant amount of literature on migrant women’s experiences of GBV focuses on women’s perceptions of violence and their help-seeking processes. These studies broadly indicate unique barriers that prevent migrant women from disclosing their experiences and seeking support. Certain cultural and religious norms and practices, isolation/alienation, loss of informal supports, migration/acculturation distress, language barriers, lacking adequate information regarding laws, structures, and services, distrust of state authorities, having precarious migration status, or fear of deportation are underlined as some primary obstacles for women’s disclosure of violence and help-seeking (Ahmad et al., 2009; Alaggia et al., 2012; Aujla, 2021; Holtmann and Rickards, 2018; Hulley et al., 2023; Okeke-Ihejirika et al., 2020).

Despite the reported complexity and multiplicity of the barriers that migrant women may experience, critical policy scholars highlight the difficulties in conceptualizing the relationships between culture and GBV in research and practice (e.g., Abraham and Tastsoglou, 2016; Freedman et al., 2022a,b; Jayasuriya-Illesinghe, 2018; Jiwani, 2005; Sokoloff and Dupont, 2005). These scholars mainly criticize the tendency to rely overwhelmingly on a form of culturalist essentialism to explain migrant women’s experiences with GBV in academic and public discourses. Jiwani (2005) argues that culturalist approaches, which reduce GBV among migrant communities to a cultural problem, often result in “the production of cultural prescriptions that further entrench stereotypic representations of particular ethnic groups,” leading to further marginalization of migrant GBV survivors within and outside of their communities. Jayasuriya-Illesinghe (2018, p. 344) similarly underlines how migrant GBV survivors are often blamed and depicted as “those who uphold traditional cultural values.” This misapprehension simplifies and homogenizes migrant women’s experiences of and responses to GBV by creating a false hierarchy between “the West and the Rest” (Yuval-Davis, 2015, p. 98) as “non-violent” and “violent” cultures, which contributes to the ongoing marginalization of women belonging to racial and ethnic minority groups by reinforcing existing social barriers and inequalities that they face in various aspects of their lives.

The predominant use of cultural frameworks also results in overlooking structural determinants in analyzing the risks and vulnerabilities experienced by migrant women and girls (Jiwani, 2005). As put by Freedman et al. (2022a, p. 8), this diverts attention from “fundamental causes of gender-based violence rooted in unequal, racialized and gendered structures of domination and control in host countries.” In this way, government policies that create conditions furthering systemic inequalities and discrimination experienced by migrant groups (e.g., poverty/unemployment, economic exploitation, service gaps, accessibility issues, social exclusion, racism/sexism) remain invisible (Jayasuriya-Illesinghe, 2018). Therefore, when it comes to studying migrant women’s help-seeking experiences, rather than solely relying on cultural frameworks, an analysis of how cultural-and family-level barriers interact with systemic and structural ones gains critical importance (Sokoloff and Dupont, 2005).

## State responses to GBV and migration in Canada

GBV continues to be a crucial problem in Canada, disproportionately affecting women and girls. Considering Canada’s growing migrant and newcomer population, male violence against migrant women and girls remains a central concern (Okeke-Ihejirika et al., 2020). While Canada has some legislative strategies and policies addressing GBV and migration (e.g., “It’s Time: Canada’s Strategy to Address and Prevent GBV” and “Immigration and Refugee Protection Act”), it is argued that existing state strategies remain limited in successfully responding to GBV, particularly for women and girls in marginalized communities including migrants and refugees. This limitation is believed to contribute to further structural/institutional (re)production of GBV experiences (Abraham and Tastsoglou, 2016). In this sense, overdependence on criminal justice frameworks in centrally shaping state responses to violence, lack of intersectional understandings of GBV in legislation and policy documents, the failure to address its structural and societal roots, and neoliberal tendencies in current migration and service provision policies are suggested to create complex challenges and barriers to migrant women’s safety from GBV (Alaggia et al., 2012; Abraham and Tastsoglou, 2016; Jayasuriya-Illesinghe, 2018; Okeke-Ihejirika et al., 2020; Singh, 2010; Tabibi et al., 2018; Tastsoglou et al., 2022).

State legislation and policies in Canada are generally criticized for not efficiently recognizing and addressing the social, economic, and political determinants of GBV. This is argued to result in “tunnel vision” (Abraham and Tastsoglou, 2016, p. 576), whereby the problem of violence is individualized, depoliticized, and/or culturalized as is the case for ethnic-racial minority migrant women. For instance, many migrant women in Canada are shown to experience a downward socioeconomic mobilization in the host countries (e.g., not being able to practice their profession, remaining unemployed for an extended period after resettlement, living in poverty, experiencing increasing economic dependence on their spouses) (Alaggia et al., 2009; Jayasuriya-Illesinghe, 2018; Tabibi et al., 2018). While economic vulnerability is a crucial factor that would increase women’s susceptibility to GBV and limit their chances to disclose violence and seek help (Ahmad et al., 2009; Alaggia et al., 2009; Aujla, 2021), such challenges are often reported as overlooked under the neoliberal migration policies (Jayasuriya-Illesinghe, 2018, p. 340).

Intersecting with the class-based barriers, systemic inequalities and discriminations are underlined as still significantly affecting the accessibility of supports and services in Canada for migrant women, especially those who are racially and ethnically diverse (Alaggia et al., 2012; Tabibi et al., 2018; Tastsoglou et al., 2022). Inadequate language translation services, for instance, are considered a major example of unequal and unjust treatment of migrant and refugee women, making existing services inaccessible and unavailable to them (Tastsoglou et al., 2022). Studies also indicate that previous experiences and/or fear of discrimination and racism primarily prevent racial-ethnic minority migrant women from accessing services (Okeke-Ihejirika et al., 2020; Tabibi et al., 2018; Tastsoglou et al., 2022). In addition, limited cultural competency in service provision is highlighted as another crucial structural barrier contributing to migrant women’s further marginalization while seeking support (Tastsoglou et al., 2022). One-size-fits-all solutions primarily based on Westernized “rescue and prosecute” (Okeke-Ihejirika et al., 2020, p. 789) strategies without considering diversities in women’s experiences, perspectives, needs, and expectations, are underscored as core contributors to decreasing women’s likelihood of accessing and benefiting from the available services (Abraham and Tastsoglou, 2016).

Insufficient and inconclusive statistical data on the prevalence, correlates, and determinants of GBV among migrant women in Canada is also highlighted as a crucial factor resulting in the inadequate representation of their experiences of GBV (Alaggia et al., 2009; Jayasuriya-Illesinghe, 2018; Okeke-Ihejirika et al., 2020; Tabibi et al., 2018). The findings from the 2018 Survey of Safety in Public and Private Spaces (SSPPS), for instance, reveal lower rates of lifetime occurrence of intimate partner violence among minority women, including migrant women (Cotter, 2021). However, as with previous survey studies, these results remain inadequate for several reasons. First, the survey was conducted only in English and French, which increases the likelihood of the exclusion of migrant and refugee women with limited language competency. Second, the major proportion of minority women who participated in the survey was reported as including younger, highly educated, and employed women. This is a crucial limitation, particularly considering the reported lower rates of labor force participation and the higher prevalence of unemployment and low-income status among migrant women (Hudon, 2015). Furthermore, as the SSPPS only included women who live in “households,” the results do not reflect the experiences of women living in institutions, including shelters and women experiencing homelessness (Cotter, 2021). However, according to the Survey of Residential Facilities for Victims of Abuse (SRFVA) in 2020/2021 (Ibrahim, 2022), “non-permanent resident” women and women without English/French competency are overrepresented among groups in the facilities, and nearly 30 percent of residents are racial and ethnic minority women (e.g., Asian, Chinese, Latin American, African, or Caribbean). Thus, such general national surveys in Canada, like the SSPPS, seem inadequate in representing issues experienced by migrant and refugee women, which makes them “less visible in public and political spaces” (Jayasuriya-Illesinghe, 2018, p. 343).

## Methodological considerations

The current study is based on a Canadian University-based research program that is part of the international research project, *Violence Against Women Migrants and Refugees: Analyzing Causes and Effective Policy Response*. The latter consists of seven country-based research teams and aims to understand better how patterns and dynamics of violence against women are being shaped within migration contexts across countries and make policy recommendations to improve migrant and refugee women’s access to support and services. In line, GBV-MIG Canada examines how migrant and refugee women experience GBV, the institutional contexts in which such violence occurs, what strategies they develop to protect themselves, and what they experience through their interactions with services.

The Canadian project includes several interview-based data sets, with “key informants,” “front-line workers,” and migrant and refugee women (MRW) of a variety of legal statuses, but also secondary interview-based data sets with MRW. The present article derives from a secondary data set collected for a five-year project, the Canadian Domestic Homicide Prevention Initiative with Vulnerable Populations (CDHPIVP), which includes interviews with survivors of severe domestic violence. CDHPIVP study broadly investigates diverse risks and vulnerabilities that women survivors of GBV encounter, barriers and challenges to their safety-seeking, and their experiences with GBV-related services and supports (Straatman et al., 2022). The analysis that follows is based on 17 interviews with migrant women from this project, with permission of the co-PIs and Research Ethics Board approval of the academic institutions involved.

Participants were recruited through a combination of public outreach efforts, including press conferences, project events, posters, and social media posts. The participants were migrant women from four different regions in Canada, Ontario, the Prairie provinces, Quebec, and British Columbia. Three participants reported that they had been subjected to their fathers’ violence while growing up and had witnessed violence from their fathers toward their mothers. All other participants were exposed to violence by their husbands, common-law partners, or dating partners. Most participants reported migrating as dependents to accompany their husbands, while others relocated with their parents, and several cited economic reasons as the primary motivation for their migration. While for some participants, violence had started once after they moved to Canada, most of them reported that it had started while they were in their home countries and continued/aggravated after their migration. All women except five of them had at least one child. The participants were from diverse regions, including North Africa, South and East Asia, the Middle East, Western and Eastern Europe, and the Caribbean. The participant age range was from 20 to 65 years. All participants were highly educated, and most were employed-except four of them, whose employment information could not be obtained. The time of their residency in Canada ranges from three to 45 years. All were either permanent residents or obtained their Canadian citizenship during their migration process.

### Interview procedures for the secondary data set (CDHPIVP)

For the CDHPIVP project, individual, semi-structured interviews were conducted between August 2019 and December 2022, either by phone, online, or in person. They lasted from one and a half to 3 hours. All interviews were audio-recorded and transcribed verbatim. Each participant was given a pseudonym. Qualified researchers (mostly graduate students, research associates, and post-doctoral researchers) were recruited and further trained to conduct the interviews. Having adequate experience in qualitative interviewing and previous experience of working with trauma and GBV were primary criteria for the interviewer recruitment. Interviews were conducted in a narrative storytelling form based on an interview guideline. Participants were asked to tell three stories based on their lived experiences where they feared for their physical and/or emotional safety and tried to protect themselves. Open-ended questions were asked to explore different aspects of the stories shared. These questions included what actions participants took to ensure their safety, what their needs were to feel safer, what was helpful to them or what was not, or what helpful or unhelpful reactions and responses they encountered when/if they sought support.

### Data analysis

The current study employed a feminist approach to data analysis, focusing on the interpretive and reflexive exploration of the lived experiences of migrant women. Our analysis aimed to offer a situated and survivor-centered understandings of the GBV-migration interaction. Compatible with this approach, we used a reflexive thematic analysis to systematically code the interviews (Braun and Clarke, 2006), through which we explored and identified the shared and divergent processes, themes, patterns, and meanings in our participants’ narratives. We followed a six-step coding and analysis strategy. The first phase of the analysis involved a familiarization process with the interviews, where we read the interviews without going into systematic coding, made initial observations, and drew preliminary insights. The following two phases involved systematic coding of the transcribed narratives through a qualitative analysis software program, QDA Miner. First, semantic/descriptive codes were developed based on the processes and events told by the participants. This step was followed by developing more conceptual codes based on our analysis of the patterns, frequencies, and meanings that emerged through the previous coding phase. These conceptual codes were used in the following steps to organize the data around central themes by defining their boundaries and specific features. The reviewing of the generated themes follows this phase to see if each has a particular focus, conveys a distinctive meaning, and is relevant to the research objectives. The last step of the analysis, described as the writing phase by Braun and Clarke (2006), aimed to ensure that the data was represented cohesively and coherently in the final analysis and writing.

## Findings and discussion

Three core themes, “migration as a tool for further violence by offenders,” “migration as a cultural stigmatization process,” and “struggling with an unaccommodating system” were identified in the participants’ narratives. These themes primarily address women’s post-arrival experiences regarding (i) how the migration process, as particularly manipulated by the offenders, intensified their risks and vulnerabilities for GBV, (ii) how their safety options were restricted due to the barriers and stigmas they experienced within their communities, and (iii) how formal services were mostly inaccessible to them and they encountered a highly disconnected system when they sought formal help.

### Migration as a tool for further violence by perpetrators

As we conceptualize the migration context as a critical determinant of women’s experiences of safety-seeking in violent relationships, we first examined the data by asking how the process of migration shaped their vulnerabilities. Our analysis illustrated that the migration process was strategically manipulated by violent partners to victimize women further. It was rare that the violence started with migration; in most cases, it intensified violence that women had already been experiencing. Our participants frequently described how perpetrators used migration-related processes (e.g., preventing women from applying for permanent residency or citizenship, seizing women’s migration documents, and isolating women from their communities) as a form of control. For example, the following quote by Alisha, a South Asian woman in her 40s, illustrated how preventing women from applying for Canadian citizenship could become a powerful control tactic:

… that was one of his control things. Everybody, him and the boys, have their Canadian citizenship, except me. He didn’t allow me-that was one of his forms of control. So, I renewed my PR three times now every five years, I had to renew it.

Similarly, Emily reported that her social security card and migration-related documents were seized by her partner, which appeared to increase the isolation she experienced in the community and hindered her process of securing full-time employment:

He took my SIN card and refused to give it back… he also took my citizenship card, so when I had to get it replaced, but in order for me to get my citizenship card replaced, I had to have my landing papers so I—so for three years—this took three years for me to replace my migration papers. And so, for three years, I was basically a non-person in the community… All my—like it was to the point that my job had… they couldn’t hire me permanently because I didn’t have the stuff. (40s, from Great Britain)

These experiences (e.g., being prevented from accessing their migration documents or applying for Canadian citizenship) highlight forms of violence unique to migration contexts and crucially reveal how the policies and laws regulating the rules and practices for acquiring residency or citizenship can prepare the ground for these forms of GBV (Sahraoui and Freedman, 2022; Sisic et al., 2024; Tan and Kuschminder, 2022). While these violent behaviors can be broadly defined or categorized as “coercive control” or “psychological violence,” they also significantly reveal how gendered harm can be enacted in the lives of migrant women in quite distinctive ways compared to non-migrant women. In this sense, being exposed to such distinctive behaviors of abusive control seems to create a vicious cycle in migrant women’s lives, a “continuum” of precarity and GBV (Tastsoglou, 2022), where their legal/migration status lays the ground for GBV and GBV, in turn, exacerbates their already existing vulnerabilities (e.g., employment insecurity or disruption to one’s sense of community belongingness). Additionally, legal/migration status, as a structure created by laws and policies that fall short of protecting migrant women, demonstrates a different continuum of violence in which institutional/structural gaps in protection enable the perpetration of interpersonal forms of violence (Tastsoglou et al., 2021).

Controlling women’s relationships with their families back in their countries of origin, especially after arriving in Canada, or exploiting women’s financial resources during their migration journeys were also identified as common forms of GBV in migrant women’s stories. For Grace, the violence did begin after migration, and by preventing her from communicating with her family in England, her partner made sure that she had “nowhere to go”:

He knew I had got nowhere to go, and he didn’t start getting abusive until after we were in Canada, once he’d cut me off from all of my family and all of my supports… I didn’t have contact with them from the moment we got into Canada for twelve years. (50s, from Great Britain)

Jada’s experience below illustrates how the migration journey she had (including pre-and post-arrival processes) enabled her violent partner to abuse her financial resources by not allowing her to access their savings:

for those years when I was working, we have a joint account and I was sending money (from the Caribbean to Canada)… So, when we came here, I asked him about the joint account… He said, “Which account?”… I never saw the money in that account. I sent money for years, but I never saw the money (60s, from the Caribbean)

Financial control or social isolation can be experienced by any woman, regardless of whether they are migrant or not. However, what we see in these examples is how migration-related factors or specific circumstances enabled by migration processes may further amplify perpetrators’ control and dominance over women. This finding aligns with a context-specific vulnerability approach (Reilly et al., 2022), which suggests that migration contexts often aggravate GBV-related risks and threats encountered by migrant women. Overall, echoing previous studies (e.g., Hulley et al., 2023; Holtmann and Rickards, 2018; Sisic et al., 2024; Tastsoglou et al., 2021; Tastsoglou, 2023; Tan and Kuschminder, 2022), our analysis shows how the abusive exploitation of migration circumstances (e.g., legal status/documentation, economic precarity, social isolation) in the context of GBV generates a continuum of vulnerability in migrant women’s lives, further entrenching them in violent relationships and limiting their “space for action” (Harris and Woodlock, 2019, p. 534).

### Migration as a cultural stigmatization process

While describing their experiences of violence, most women identified problematic and harmful community responses when they disclosed their experiences of GBV to people within their communities. The women interpreted these community attitudes as stemming from the cultural normalization of violence against women and emphasized how discouraging and isolating it was for them to encounter such responses in their efforts to seek help. For example, Ada, a young woman in her twenties from a Muslim community, shared her observation that family violence has never been addressed and problematized in her community. She also stated that her religious identity became a barrier to moving away from violence:

I’m Muslim, so one thing that was used against me a lot was religion… I think that the problem is these topics like domestic abuse are never addressed in certain communities… no one really talks about how domestic—like how child abuse and domestic abuse—domestic abuse and then children being present during that like how serious it is.

Some women also highlighted that male-dominated norms and values in their cultures were the root cause of GBV. Nadia, a South Asian woman who had been exposed to severe psychological and physical abuse by her ex-husband, reported how cultural gender-based expectations for women in her community intensified her vulnerability to GBV:

I got educated in Canada… but then I got married in Pakistan, the arranged marriage. And I do feel there is a big culture clash that had happened, cause like, my community demands that a woman be submissive to her husband, she’s expected to obey his demands… his demands was that he expected everything to be perfect 24/7… he would get angry over like very little things.

Several women also identified the cultural normalization of violence as one of the primary barriers to recognizing it and seeking support. A South Asian woman in her 40s, Farah, explained how patriarchal expectations from women in her culture created silence around GBV and led to further isolation of women within their communities:

I come from a culture very much where the man is dominant… it’s really sad, but in my home country, there were lots and lots and lots of situations where we knew, like women who were being abused and all that, but nobody ever got involved… that’s what happens within their four walls.

Informal support from family, friends, or community plays a critical role in migrant women’s experiences of safety and protection, particularly given the structural barriers they often face in accessing formal resources, such as language barriers and the inaccessibility or inadequacy of those services (Wachter et al., 2021). However, in their efforts to disclose violence and seek help, some of the participants said they were refused help by the people they sought help from. Alisha, a Muslim woman, revealed how she felt isolated in Canada because of the language barrier and because she felt turned down by her community, as they advised her to be “patient”:

When we moved to Canada, I became more isolated, you know, I’ve got little English, it was kind of isolating… And I did find some community there, but… they would just tell me to be patient… when I did confide with two or three sisters, you know, everybody was saying like “compared to some of the men, he’s ok” or “just sabr” (meaning “be patient”)… That’s where I felt more entrapment… there was another incident where actually I met with an Imam after I left to get my Islamic divorce - where the Imam basically validated him. So, he was more emboldened.

Not being believed by their families, friends, or neighbors and encountering their indifference toward their suffering were commonly reported as holding the women back from seeking further support. This seems to limit their chance of escape and, intersecting with structural and institutional barriers, reinforces the continuum of violence in their lives. The following participants shared how their families negatively reacted when they disclosed violence, did not believe them, and pressured them to “make their marriages work”:

I shared with my sister, and she just got angry with me and didn’t believe me… she firmly believed that I had to make it work… it is very difficult as a woman when you’re isolated, you have no family or friends or support to get out of a bad situation like I was in. (Joanna, 60s, from Europe)

I told my dad, and he said, “Well, try to make it work” or something like that… He said if you want to come back here, people will laugh at you… I didn’t say anything else… I didn’t feel like I had family support, so I was pretty much going through this on my own (Jada, 60s, from the Caribbean)

These findings illustrate how the participants perceived their cultures intersecting with the racial and religious norms shaping the gendered power hierarchies and their experiences of GBV. The instances shared by our participants crucially reveal the links between normalized, everyday aspects of gendered harm (e.g., gender role expectations, male dominance and control, prioritization of “family” over women’s safety) and more severe, visible forms of violence (e.g., physical violence). Our participants reported that they were culturally expected to accept male control in their lives, tolerate violence from their partners, be patient, and deal with it privately without disclosing it to others. These expectations rendered their experiences of violence and suffering invisible, leaving them further isolated and without help. Thus, when families and communities endorse men’s violence and refuse to intervene, this inaction becomes part of the cumulative harm women are subjected to throughout their life cycles. This aligns with what Tastsoglou et al. (2022) calls “violence by omission”—addressing incremental or ongoing harm in the lives of migrant individuals caused by systemic institutional indifference to their need for help. It can be considered as a continuum of interpersonal and institutional violence as lack of inadequate protection exacerbates vulnerability to GBV in their interpersonal contexts (Tastsoglou et al., 2021). In this sense, our analysis demonstrates that the women’s social networks and their communities may also (re)produce GBV and exacerbate women’s struggle by remaining indifferent to their suffering and leaving them without protection, thus becoming part of the continuum of violence.

While addressing patriarchal norms and values intrinsic to their cultures as part of the continuum of violence they were subjected to, some of the women, particularly Muslim women, also spoke about a “double bind,” where they feared encountering the stereotypical Westernized ways of thinking about violence in migrant communities. They expressed that the overemphasis on culture in migrant women’s experiences of GBV could lead to further stigmatization of them. Thus, the expectation of such stigmatization and/or experiences of it seemed to hinder women’s efforts to seek help. For example, Saneem, a North African Muslim woman who migrated to Canada nearly 25 years ago, described her perceptions regarding stereotypes against migrant communities:

All families experience violence, whether they are Canadians or migrants or whatever… but it seems that when you're an immigrant family, it's exacerbated. Look at their culture!… but no! It was just… violence is in men whether they are from one culture or another there!… I wouldn't say it's the culture, it's the men, period.

In the following examples, Alisha and Ada also highlighted how their fear of being stigmatized as Muslim women became a barrier for them to disclose violence and seek support:

I was the only hijabi there [in her workplace]… they would be like “oh poor Muslim woman”… That stigma that was the thing… communicating with immigrant communities… They have that “oh, in this country when you come, we don’t kill women”… making them feel they are barbaric, right? (Alisha, 40s, from South Asia)

I also felt like I didn’t want to perpetuate the stereotype that Muslim women are victims of abuse… for some reason, if I tell this to anyone who’s Caucasian, I might be perpetuating a stereotype because they wouldn’t understand like they just wouldn’t understand how I feel about this. (Ada, 20s, from North Africa)

Jiwani (2005) highlights that “singling out particular cultural communities and suggesting they have a proclivity to violence” (p.852) results in further stigmatization and marginalization of migrant communities. Our participants’ accounts suggest that the fear and anticipation that their culture, communities, and themselves would be stigmatized or criminalized by the dominant Western culture may hinder ethnic and racial minority GBV survivors from disclosing violence and seeking help. These experiences also correspond to a “paradox” of simultaneous visibility and invisibility of GBV in migrant women’s experiences (Jayasuriya-Illesinghe, 2018). For racial and ethnic minority women, the cultural background they come from, as well as their migrant identities, become highly visible in the context of GBV. However, at the same time, when GBV is reduced to a cultural problem, their experiences are disregarded and made invisible; they often face the risk of being blamed, undermined, or misrepresented, and thus become less willing to acknowledge or disclose the occurrence of violence in their lives (Freedman et al., 2022b; Jayasuriya-Illesinghe, 2018; Jiwani, 2005). In a sense, structural deficiencies in addressing and responding to GBV in migrant women’s experiences, particularly related to their marginalization through the culturalization of their experiences by the very structures they seek help, create significant barriers to seeking support and reinforce the continuum of violence.

### Struggling with an unaccommodating system

The third core theme shows how participants described their processes of reaching out to formal supports, focusing on both their accessibility and the interactions with formal support structures in Canada —particularly their interactions with the police, courts, and health care system-. The following subsections, “inaccessibility of information and resources” and “interactions with the formal support services,” highlight how our participants experienced formal help-seeking and what systemic challenges and barriers they encountered. These experiences reveal that the systemic failures in providing protection —within institutions and structures such as the court system, healthcare, police, and GBV services— translate into migrant women’s interpersonal contexts. In other words, GBV is enacted in structural and interpersonal contexts, with these intersecting (Tastsoglou et al., 2021; Tastsoglou, 2023).

#### Inaccessibility of information and services

The following examples show that accessing the correct information is not straightforward. Most women emphasized that finding the most relevant and reliable sources of information was a challenging task:

Well, there’s not a lot of information out there, even in the wide world of google… on how to do this, how to navigate a situation like this… On internet there’s twenty million numbers you can call but… the thing is too that people who are in these situations are so stressed… and doing all this kind of research… it's just not easy to access information and the correct information. I don’t have hours to sit on my phone. (Elena, 40s, from Europe)

I think a big part of it is exactly the knowledge… Like you research things, but then you don't really find things that could help you. I researched domestic violence, but I didn't find much that could support me, I think. (Noor, 30s, from the Middle East)

Alisha similarly described how she experienced the gaps in the system:

… because I saw there was a disconnect between the social services… after my whole experience, I was trying to do like a road map of how to escape because there were gaps. And I went to one agency. They didn’t tell me that I have to do this, or I had to bring this… There should be… like a clear handout on this is… because you’re thinking, “oh my god, where am I going to – how am I going to deal with stuff?”

Previous research indicates how it can be challenging for migrant women to navigate their way to safety in the absence of a clear and well-defined “road map” about the available resources and processes that can guide their steps to move away from violence (e.g., Barrios et al., 2021; Yalcinoz-Ucan et al., 2024). While non-migrant GBV survivors can also encounter these challenges, when it comes to migrant women, such challenges are experienced in multilayered ways due to various factors such as language barriers, social isolation, or unfamiliarity with the systems and services (Acquadro-Pacera et al., 2024; Holtmann and Rickards, 2018; Okeke-Ihejirika et al., 2020; Murugan et al., 2023; Rubini et al., 2024). Irene, for example, a survivor of family violence by her father, reported that, due to her parents’ undocumented status, she had no information about and no access to available healthcare services in Canada:

I didn't even know I had a health insurance card until I was 18… we didn't have access to health services. There would have been no doorways to talk about it. Especially since my mother was, my parents didn't have their papers… it was out of the question to go see a doctor. (20s, from Eastern Europe)

Elena similarly stated that she did not know that she could reach out to her family doctor to seek help regarding her experiences of GBV:

When I was in the shelter, I was told a lot that I should have called my doctor. I did not know this… I knew I could to my doctor for depression and certain things, but I didn’t know if I was in an abusive relationship, I could go to my doctor, and my doctor would be able to take steps to help me. (40s, from Europe)

Such information gaps seem to have systemic roots and aggravate the invisibility and silence around migrant women’s experiences of GBV (Rubini et al., 2024; Wachter et al., 2021). This, in turn, as shown in the experiences of our participants, leads to the exclusion of migrant women from protection and services (Jayasuriya-Illesinghe, 2018).

Several of our participants also emphasized that other intersecting aspects of their identities besides being migrants aggravated their difficulties in accessing information and support. Having disabilities, living in a rural environment, and/or being older, in interaction with migration and GBV dynamics, seemed to create complex circumstances of vulnerability in their lives. Joanna, for instance, emphasized that her circumstances continued to be challenging, as a migrant woman in her 60s with disabilities and had limited knowledge about the available resources:

I’m still in a fairly vulnerable position at this age because of all of the ongoing abuse I have four different disabilities, and uhm, so it’s very challenging. I’m also an immigrant, so I’m not fully aware of all the uhm services available in helping work here in Canada, so that was another situation that made me more vulnerable.

The following narrative also showed that having physical disabilities could restrict women’s options and prevent them from benefiting from shelter assistance:

I called the shelters… When I told them I have a visual impairment, “Oh no, but it's not accessible because this and that… and do you think you'll be able to?” Listen, for me, it's… to close the door completely! Just telling me… “Well… are you going to be able to manage?… are you going to be able to do like all the other women?” What is that? Like all the other women?… That's already closing the door! (Saneem)

Another woman also underlined that living in a rural area where there were no resources or services for GBV survivors and/or migrants, intersecting with limited employment and housing options, became a critical barrier for her to reaching out for supports or planning escape:

There was no plan in place for employment or anything like that or housing. So, while we were… it was a really small town. And there weren't very many resources available in the town for immigrants… Immigrants, period. But also for domestic violence. And I wasn't really able to receive any kind of help there, formally (Alisha)

These examples demonstrate how women’s vulnerabilities overlap based on their multiple, simultaneous identities and how the systemic response remains inadequate in designing and providing services that are tailored to their diverse and complex needs. Therefore, in the context of women’s intersecting identities and the consequent inequalities they face, this systemic negligence —interacting with GBV dynamics in their interpersonal settings— seems to generate “cumulative” harm and suffering in their lives.

Correspondingly, some of our participants underlined how “one-size-fits-all” options or services offered did not work for them, leaving their needs and expectations unheard or unrecognized. They specifically criticized the shelter stay being presented as the sole option or solution:

The only thing that they [the police] gave me was the shelter phone number. But I didn't need shelter. I actually just needed to feel safe. I think… they kind of telling me what to do to feel safe (Noor, 30s, from the Middle East)

the resource sheet that they [the police] gave me was interesting to me… it was really not… accessible for me… if you want to leave home, you have the shelters. Period. But… if I want it to be settled differently, there was no… there was nothing of… of that. (Saneem, 50s, from North Africa)

Services are often offered in generic and decontextualized ways without considering how different women experience GBV differently and, thus, have a range of diverse needs. Therefore, from an intersectional perspective, these experiences highlight the crucial need for “context-specific” and nuanced approaches in system responses to GBV survivors’ needs, especially for those with multiple marginalized social positions (Abraham and Tastsoglou, 2016; Cardenas, 2020; Reilly et al., 2022; Tan and Kuschminder, 2022).

#### Interactions with formal support services

The analysis demonstrated that, in many instances, formal support responses were experienced as uninformed, inadequate, and ineffective. When disclosed violence and sought help, most of our participants reported that their stories were ignored or trivialized, and, at times, their experiences of violence were questioned. We consider these experiences crucial in revealing the links, or continuity, between women’s experiences of GBV in their interpersonal contexts and the structural forms of violence they encountered.

#### Healthcare responses to GBV

The analysis showed that, for most of our participants, it was challenging to receive proper and much-needed help from healthcare services. The narratives demonstrated how warning signs and possible risks could be overlooked by practitioners. Some women emphasized that no domestic violence risk assessment had been conducted by healthcare professionals. Olivia, who experienced a significant weight loss over a short period, emphasized that she had never been asked any question about domestic violence, which limited her chances of disclosing violence and seeking support:

No one in the health care ever asked me if I was safe at home. I was sick, I lost like… my normal weight is like 135, 140, and I got down to like 115, over like 3 months. And no one ever said, “Are you safe at home?”, “what’s going on?” And I didn’t tell. If someone had asked, I might have, if someone had asked me. (40s, from Europe)

Some women also stated that even when they directly disclosed their experiences of violence, they did not receive any support from their healthcare practitioners. Anna, for example, reported that other than treating her physical injuries, the doctor and nurse she interacted with did not follow any risk assessment procedures or provide further assistance (e.g., informing her about available GBV services or making referrals to victim services):

The doctor asked [about the injury]… And I said “Well, my husband pushed me, and I hit my hip.” And he’s like, “Wow… that’s unfortunate.” And that was it… Nobody asked anything… No questions. They had to remove this bloody part of my hip. And stitched it and let me go. (Anna, 40s, from Eastern Europe)

The following example similarly shows how women’s concerns can be entirely minimized by healthcare practitioners:

I tell the pediatrician - I say, "Look, I've been a victim of domestic violence. My son was a witness."… And she says to me, and I'm stunned, she says, "Listen, ma'am, this is a conflict between you and the man, it doesn't concern the child… So, I write her an email, I say, “I'm not happy at all, this, that.” She never wrote me back. (Camilla, 30s, from Europe)

In migration contexts, when there is a lack of supportive community networks, experiences of social isolation, and limited knowledge regarding formal support mechanisms, women’s interactions with healthcare practitioners may become a crucial first step in identifying and assessing their risks and developing an informed safety plan. However, echoing previous research (Acquadro-Pacera et al., 2024; Rubini et al., 2024), these uninformed or dismissive healthcare responses faced by our participants compounded their vulnerability to GBV, increased their sense of isolation, and further discouraged them from seeking support. Thus, encountering these healthcare responses seemed to become a particular “point of interface” for GBV with precarity (Tastsoglou et al., 2021) in our participants’ lives. Structural inequalities in accessing healthcare and the ineffectiveness of healthcare responses to GBV exacerbate the vulnerabilities of migrant women, illustrating the continuum between precarity and GBV as experienced by our participants.

#### Interactions with the police

Our participants’ experiences with the police included many examples where they felt intimidated and disregarded. Such responses by the police were emphasized as emotionally overwhelming and re-traumatizing, as well as creating further risks to their safety. The following quotations, for instance, illustrated that psychological violence (e.g., ongoing threats and verbal harassment of ex-partners, especially after divorce) was not considered “severe enough” to warrant intervention:

I've reported him about the emotional abuse… or manipulation. And basically, the law is that… Basically, it was like, it was not severe enough. (Olivia, 40s, from Europe)

The police here do not… the culture is to not deal with it… Because I've had several interactions with them since I left him, that clearly shows a pattern of him harassing me with the system. I even went to the victim services person at the police department and was like, these are the e-mails that I get all the time… basically, she is like, “Well unless he’s threatening to harm you, there's really nothing we can do.” (Emily, 40s, from Great Britain)

Aligning with the continuum of violence framework, these experiences are quite critical in showing how the police responses mainly focus on physical violence, and when it comes to psychological violence, such experiences are more likely to be dismissed or not taken seriously. These experiences can be understood with what Abraham and Tastsoglou (2016) call “tunnel vision,” where GBV is primarily viewed through the lens of criminalization, causing its broader conceptualizations and definitions to be overlooked in systemic responses.

The following quotation also exemplifies how interaction with the police could be intimidating for some women, including cases where they felt stigmatized and derogated by the police due to their migrant backgrounds:

I was afraid to talk to them when they asked me questions… I didn't feel safe with the police… they have a speech… their body… language is scary… and you saw in their eyes that they were saying to themselves, “Oh, it's an immigrant family”… they talked to me in a… very arrogant way… I felt… as if I was a… lesser person who is a battered woman who accepts violence. (Saneem, 50s, from North Africa)

This can be seen as a form of institutional violence, revealing how GBV can be perpetuated or (re)produced through women’s interactions with the system. The police’s stigmatizing response appears to limit access to protection. In a way, as perceived by the woman, being an ethnic minority migrant woman who experiences domestic violence fits into and reinforces a certain stereotype of victimhood and vulnerability held by the police —a stereotype portraying migrant women, especially those who are Muslim and/or racial-ethnic minority, as helpless “victims” of a “barbaric” culture. This stereotype not only undermines migrant women’s agency by also misconstrues GBV as a culture-specific phenomenon in a socio-political context impacted by islamophobia, xenophobia, or racism (Abraham and Tastsoglou, 2016; Jiwani, 2005; Tastsoglou et al., 2021).

#### Going through the family court system

The judicial system was experienced as very unaccommodating, in which the women struggled to ensure their safety and wellbeing. In fact, the interviews revealed that, in some cases, going through the family court processes resulted in further harm and reinforced women’s vulnerability. Rather than the system protecting and supporting them, several participants highlighted that the system worked against them, and they had to “fight” it. Jada’s narrative below illustrates how such experiences critically jeopardized her safety and mental health:

Things were so uhm bad at the time in terms of I felt like so unsupported by that whole system… it was going through the court system that drove me to the point where I literally contemplated suicide… I just said you know what I have to fight the system, the system that’s supposed to protect victims. (Jada, 60s, from the Caribbean).

Some women highlighted that the perpetrators used the family court system as a tool for further violence after separation. Issues related to custody arrangements and divorce settlements were commonly addressed as creating significant struggles. Grace, for example, pointed out that the court’s stance of neutrality, which did not acknowledge the existence of GBV, felt unjust and crucially undermined the violence she had experienced:

This children’s lawyer never mentioned child abuse in his presentations to the court… I brought it up, but the judges wouldn’t listen, and I felt awful. The court was trying to be so neutral. What they did, in fact, they put me and my ex on the same scale like it’s both your fault, you both guilty. You both, you’re equal… and I was telling them I cannot be held equal to my abuser (Grace, 50s, from Great Britain)

While feeling intimidated during the court processes, particularly when interacting with lawyers and judges, was identified as a common experience among several participants, a few also shared instances in which they were openly disbelieved and discriminated against:

When I left, I took everything out, I put it all in closets… This is a judge who told me

… "For someone who says she's being abused, you've done a good job of preparing to leave.” That shocked me. That judge, I filed a complaint against her. (Camilla, 40s, from Europe)

I had to file a judicial complaint against the judge, and it was accepted, and she had to give me a letter of apology because I was insulted and discriminated against based on the fact I’m a foreigner. That my—I have an accent. She insulted me for my accent, and she said I understand why you don’t understand because you’re a foreigner… I was so intimidated by her. (Anna, 40s, from Eastern Europe)

These interactions with the court system —similar to the ones with the healthcare services and the police— illustrate how violence can be enacted by state institutions through discriminatory practices and/or systemic negligence. When GBV survivors encounter state-based discrimination or negligence, it becomes a significant barrier to their safety-seeking efforts and adds to their struggle by (re)producing the cycle of violence and vulnerability in their lives (Tastsoglou et al., 2021; Tan and Kuschminder, 2022). Therefore, our findings suggest a continuum, or a vicious cycle, between interpersonal GBV and institutional violence —both of which are rooted in intersecting systems of oppression or disadvantage (e.g., gender identity/sex, sexual orientation, migration status/nationality, age/disability, racial or ethnic background).

## Conclusion

This study focused on migrant GBV survivors’ processes of disclosing violence and seeking help and investigated a range of factors or processes hindering their efforts to ensure safety in their lives. Our analysis primarily demonstrated how migration contexts created unique GBV experiences and distinctly shaped women’s responses to GBV across interpersonal, community, and structural contexts. Based on the analysis of our participants’ lived experiences, we began by showing the ways in which systemic and institutional gaps, community-based barriers, and cultural stigmatization processes can become a vehicle for further violence in women’s interpersonal context and trace these experiences to the cultural and community stigmatization processes as well as systemic obstacles and challenges they encountered. Our findings demonstrated the continuum between precarity and GBV, as well as between systemic and individual forms of GBV in migration contexts. Within this continuum, precarity and GBV interact in a vicious cycle that exacerbates individual vulnerability. At the same time, systemic and structural forms of violence serve to reinforce and reproduce individual experiences of GBV.

Our research first demonstrated the processes that migration contexts increased women’s overall vulnerability to GBV. We showed how migration-related circumstances can be strategically and manipulatively used by perpetrators to exert distinct forms of control over women, limiting their options and opportunities and entrapping them in violence. Our findings also suggested that migration-related policies, laws, or practices create conditions that facilitate such abusive behaviors. At the hands of perpetrators, these mechanisms were instrumentalized to exploit further and harm women.

The findings also pointed to crucial challenges that women faced when they turned to their communities and/or families to disclose violence and seek help. Our participants’ experiences suggested a “continuum” between gender power relations embedded in their cultures (e.g., culture-based gender norms and expectations) and their GBV experiences. We showed that, in most cases, their communities abandoned the women by staying negligent toward their suffering from GBV and refusing to intervene. Most importantly, however, our analysis addressed a cultural stigmatization process where our participants feared that the focus on their cultures in construing their experiences of GBV would further stigmatize them and their communities. This misconstruing and consequent stigmatization occurs through “comparing a seemingly backward, traditional, and oppressive cultural system to the modern, progressive, and egalitarian culture of the West” (Jiwani, 2005, p. 852) —based on a created hierarchy between “non-violent” and “violent/barbaric” cultures (Yuval-Davis, 2015). This cultural stigmatization, in turn, made the migrant women less likely to disclose GBV and seek outside support.

Another critical aspect of our analysis was to reveal how the inadequacy and ineffectiveness of systemic responses to migrant GBV survivors’ experiences and needs, particularly in the cases of intersecting disadvantages based on their multiple, simultaneous identities (e.g., rural migrant women, migrant women with disabilities, older migrant women), aggravate GBV-related risks and vulnerabilities in their lives. Alongside information gaps and accessibility barriers, the findings demonstrated that the women’s interactions with healthcare services, the police, and the family court system involved many instances where they encountered uninformed, negligent, stigmatizing, and/or discriminatory attitudes and practices. These encounters, conceptualized as forms of institutional or structural violence, seemed to significantly reinforce a continuum of vulnerability across interpersonal and structural contexts in the lives of migrant GBV survivors. Therefore, this study critically demonstrates how the lack of well-coordinated and systemic state intervention further perpetuates and aggravates precarity for migrant GBV survivors.

Our findings highlight the interconnectedness of interpersonal, community, and structural contexts in intensifying precarity in migrant women’s lives. We showed how these interconnected and intersectional disadvantages and challenges lead to a vicious cycle that restricts women’s opportunities and chances to escape violence and, in turn, further aggravates their individual vulnerability to harm.

Based on these results, the study has several implications. First, given the potentially important role of community and family networks in disrupting the cycle of violence in survivors’ lives (Goodman and Smyth, 2011), it is clear that there is an urgent need to increase GBV awareness within migrant communities. This can be achieved through community-based advocacy as well as by bridging “formal and informal networks” in policy and practice (Wachter et al., 2021, p. 2371).

Second, our findings indicated the necessity of an intersectionality approach in systemic responses to GBV (e.g., service responses including the GBV-related victim services, healthcare, the police, and the judicial system) to better address and acknowledge how women from diverse backgrounds encounter intensified vulnerabilities due to their multiple and overlapping disadvantages, which locate them at higher risk of being subjected to GBV (Rubini et al., 2024; Sokoloff and Dupont, 2005; Tan and Kuschminder, 2022). Applying an intersectional lens would improve service responses to the diverse needs of women and reduce the risk of negligent and discriminatory practices toward ethnic and racial minority women.

Furthermore, our research revealed the long-term impacts of institutional forms of violence on our participants’ wellbeing and safety. Thus, we consider any systemic effort to dismantle and disrupt discriminatory ideologies and practices (e.g., racist, sexist, xenophobic, or Islamophobic) embedded in structures and services crucial. Finally, echoing previous studies (e.g., Jiwani, 2011; Sisic et al., 2024; Tastsoglou et al., 2021), our research highlights the importance of acknowledging the distinct forms of precariousness, vulnerability, and GBV experienced by migrant women in order to develop tailored prevention, intervention, and support programs.

A significant limitation of this study is that all participants were highly educated. This characteristic of the sample may reflect a selection bias inherent in the recruitment process, particularly given that the recruitment was conducted through a public open call. The homogeneity in educational background could limit the generalizability of the findings, as the study may not fully capture the diverse range of migrant women’s experiences, especially those shaped by lower levels of education or different socioeconomic contexts. Future research should strive to include participants with more diverse educational and socioeconomic backgrounds to provide a more comprehensive understanding of these issues.

## Data Availability

The raw data supporting the conclusions of this article will be made available by the authors, without undue reservation.
